# Whole body glucoregulation and tissue-specific glucose uptake in a novel Akt substrate of 160 kDa knockout rat model

**DOI:** 10.1371/journal.pone.0216236

**Published:** 2019-04-29

**Authors:** Edward B. Arias, Xiaohua Zheng, Swati Agrawal, Gregory D. Cartee

**Affiliations:** 1 Muscle Biology Laboratory, School of Kinesiology, University of Michigan, Ann Arbor, Michigan, United States of America; 2 Department of Molecular and Integrative Physiology, University of Michigan, Ann Arbor, Michigan, United States of America; 3 Institute of Gerontology, University of Michigan, Ann Arbor, Michigan, United States of America; Hospital Infantil Universitario Nino Jesus, SPAIN

## Abstract

Akt substrate of 160 kDa (also called AS160 or TBC1D4) is a Rab GTPase activating protein and key regulator of insulin-stimulated glucose uptake which is expressed by multiple tissues, including skeletal muscle, white adipose tissue (WAT) and the heart. This study introduces a novel rat AS160-knockout (AS160-KO) model that was created using CRISPR/Cas9 technology. We compared male AS160-KO versus wildtype (WT) rats for numerous metabolism-related endpoints. Body mass, body composition, energy expenditure and physical activity did not differ between genotypes. Oral glucose intolerance was detected in AS160-KO versus WT rats (P<0.005). A hyperinsulinemic-euglycemic clamp (HEC) revealed insulin resistance for glucose infusion rate (P<0.05) with unaltered hepatic glucose production in AS160-KO versus WT rats. Genotype-effects on glucose uptake during the HEC: 1) was significantly lower in epitrochlearis (P<0.01) and extensor digitorum longus (P<0.05) of AS160-KO versus WT rats, and tended to be lower for AS160-KO versus WT rats in the soleus (P<0.06) and gastrocnemius (P<0.08); 2) tended to be greater for AS160-KO versus WT rats in white adipose tissue (P = 0.09); and 3) was significantly greater in the heart (P<0.005) of AS160-KO versus WT rats. GLUT4 protein abundance was significantly lower for AS160-KO versus WT rats in each tissue analyzed (P<0.01–0.001) except the gastrocnemius. Ex vivo insulin-stimulated glucose uptake was significantly lower (P<0.001) for AS160-KO versus WT rats in isolated epitrochlearis or soleus. Insulin-stimulated Akt phosphorylation (in vivo or ex vivo) did not differ between genotypes for any tissue tested. Ex vivo AICAR-stimulated glucose uptake by isolated epitrochlearis was significantly lower for AS160-KO versus WT rats (P<0.01) without genotype-induced alteration in AMP-activated protein phosphorylation. This unique AS160-KO rat model, which elucidated striking genotype-related modifications in glucoregulation, will enable future research aimed at understanding AS160’s roles in numerous physiological processes in response to various interventions (e.g., diet and/or exercise).

## Introduction

Akt substrate of 160 kDa (also known as AS160 or TBC1D4) is a Rab GTPase activating protein and distal member of the insulin signaling pathway [1]. AS160 is a key regulator of insulin-stimulated glucose transporter 4 (GLUT4) translocation and glucose uptake [1–3]. GDP-bound Rab proteins restrain intracellular GLUT4 vesicles, and GTP-bound Rabs favor GLUT4 exocytosis. Insulin-induced phosphorylation of AS160 on selected Akt phosphomotifs inhibits GTP hydrolysis and thereby eliminates unphosphorylated AS160’s restraint on GLUT4’s intracellular localization. The relevance of AS160 for glucose homeostasis in humans was evidenced by the discovery in the Greenlandic Inuit of a common nonsense mutation is highly associated with insulin resistance and confers greater risk for type 2 diabetes [4].

The regulation of AS160 in skeletal muscle has been especially well-studied [5–16] because this tissue accounts for the greatest portion of insulin-mediated glucose clearance [17]. However, AS160 is also expressed at relatively high levels by other tissues, including white adipose tissue (WAT) and the heart [18, 19]. Insulin stimulates glucose uptake by each of these tissues, and they each contribute to normal glucose homeostasis [20, 21].

Three independent groups have created AS160-knockout (AS160-KO) mouse models to gain insights into AS160’s physiological functions [18, 19, 22]. Results from these mouse models have consistently demonstrated that AS160-KO compared to wildtype (WT) controls were characterized by: whole body insulin resistance without diabetes; insulin resistance for glucose uptake by most skeletal muscles studied; insulin resistance for glucose uptake by WAT and white adipose cells; unaltered activation of proximal insulin signaling as indicated by Akt phosphorylation in skeletal muscle and WAT; and reduced GLUT4 protein abundance in WAT and most skeletal muscles studied [18, 19, 22–24].

Although mouse AS160-KO models have provided valuable information, the reliance on a single model species has limitations. The greater size of rats compared to mice increases the amount of tissue available for analysis and facilitates some procedures that would be technically more challenging in mice. For example, we recently created and validated a method to measure glucose uptake, fiber type and abundance of various proteins in single myofibers from rats [25, 26]. Making similar measurements in much smaller mouse myofibers would be extremely difficult. We were also motivated to create AS160-KO rats because our research focuses on elucidating the mechanisms whereby exercise or calorie restriction can enhance insulin-stimulated glucose uptake by skeletal muscle. For each condition, AS160 is implicated in the elevated glucose uptake, and the amount of published research is greater for rats compared to mice. Other research questions will likely benefit from the availability of a rat AS160-KO model.

In the current study, we made a number of key measurements that were previously reported for AS160-KO mice at the whole body level (body composition, energy expenditure, physical activity, glucose tolerance test, whole body insulin sensitivity and hepatic glucose production using a hyperinsulinemic-euglycemic clamp, HEC) [18, 19, 22–24]. We also evaluated glucose uptake, Akt phosphorylation, the abundance of TBC1D1 (a TBC1D4 paralog), and the abundance of GLUT4 glucose transporter proteins in multiple tissues collected during the HEC (four skeletal muscles, WAT, and the heart), which extended results for glucose uptake during an HEC in AS160-KO mice that were made only in two skeletal muscles in a single study [19]. Measurements made in the AS160-KO rats that have not been reported in the earlier studies of AS160-KO mice included: 1) hexokinase II abundance in all of the tissues studied; 2) skeletal muscle mass; 3) skeletal muscle fiber type based on myosin heavy chain expression; 4) myocardial glucose uptake, Akt phosphorylation, and GLUT4 abundance; and 5) insulin-mediated reduction of plasma non-esterified fatty acids during an HEC. In addition, we assessed: 1) hepatic glucose production (HGP) and insulin suppression of HGP during an HEC; 2) ex vivo insulin-stimulated glucose uptake in by isolated skeletal muscles; and 3) the effect of the AMPK-activator AICAR on glucose uptake by isolated skeletal muscle. We hypothesized that AS160-KO rats compared to WT littermates would be characterized by whole body insulin resistance and glucose intolerance concomitant with lower GLUT4 protein abundance and glucose uptake in insulin-stimulated skeletal muscles, WAT, and the heart. We further hypothesized that the insulin resistance in AS160-KO rats would be accompanied by reduced skeletal muscle GLUT4 abundance and be independent of altered body composition, HGP, GLUT1 abundance, hexokinase II abundance, Akt phosphorylation or muscle fiber type.

## Materials and methods

### Materials

Reagents and apparatus for SDS-PAGE and immunoblotting were from Bio-Rad Laboratories (Hercules, CA). Anti-rabbit IgG horseradish peroxidase conjugate (#7074) was from Cell Signaling Technology (Danvers, MA). Human recombinant insulin was from Eli Lilly (Indianapolis, IN). T-PER tissue protein extraction reagent (#78510), bicinchoninic acid protein assay (#23225) and MemCode Reversible Protein Stain Kit (#24585) were purchased from Thermo Fisher Scientific (Waltham, MA). [^3^H]-2-Deoxy-D-glucose ([^3^H]-2-DG), [1-^14^C]-2-deoxyglucose ([^14^C]-2-DG) and [^14^C]-mannitol were from PerkinElmer (Boston, MA). Mouse/rat insulin ELISA kit (#EZRMI-13K) was from Millipore Sigma (Burlington, MA). Non-esterified fatty acid (NEFA) colorimetric kit (HR Series NEFA-HR) was from Wako Diagnostics (Mountain View, CA). Anti-AS160 (#ABS54) and anti-GLUT4 (#CBL243) were from EMD Millipore (Burlington, MA). Anti-phospho AS160 Thr^642^ (pAS160^Thr642^, #8881), anti-Akt (#4691), anti-phospho Akt Thr^308^ (pAkt^Thr308^; #13038), anti-phospho Akt Ser^473^ (pAkt^Ser473^; #4060), anti-hexokinase II (#2867), anti-AMP-activated protein kinase (AMPK, #2532), anti-phospho AMPK Thr^172^ (pAMPK^Thr172^, #2535), anti-TBC1D1 (#4629), anti-phospho Akt2 ^Ser474^ (pAkt2 Ser474, #8599), anti-SGLT1 (#5042) and anti-rabbit IgG horseradish peroxidase conjugate (#7074) were from Cell Signaling Technology (Danvers, MA). Anti-GLUT1 (#sc-1603) and anti-CD36 (sc-13572) were from Santa Cruz Biotechnology (Dallas, TX). Monoclonal anti-SERCA2 ATPase (#S1314) was from Sigma-Aldrich. All other reagents were from Fisher Scientific (Hanover Park, IL) or Sigma-Aldrich (St. Louis, MO).

### Animal treatment

Animal care and breeding procedures were approved by the University of Michigan Committee on Care and Use of Animals. All methods were performed in accordance with the guidelines from the Guide for the Care and Use of Laboratory Animals from the National Research Council. Rats were housed with a 12 h light/dark cycle of (lights on at 0500/lights off at 1700), at 22°C with ad libitum access to standard rodent chow (Laboratory Diet no. 5L0D; Lab Diet, St. Louis, MO) until they were fasted overnight at 1700 prior to experiments, as described below.

### Generation of AS160 mutant rats via CRISPR/Cas9

A genetically modified rat line with an *AS160 (TBC1D4)* gene knockout developed for these studies was created on the Wistar outbred genetic background using CRISPR/Cas9 technology [27, 28]. The insertion of a premature termination codon in exon 1 is predicted to result in loss of protein expression due to nonsense medicated decay of mRNA [29]. A single guide RNA (sgRNA) and protospacer adjacent motif was designed targeting coding strand: 5’ GCGACAAGCGCTTCCGGCTA TGG 3’ with a predicted cut site 111 bp downstream of the initiation codon. The sgRNA target was cloned into plasmid pX330 (Addgene.org plasmid #42230, a kind gift from Feng Zhang) as described [30].

*AS160* exon 1 targeting was tested by electroporation of Sprague Dawley (SD) rat embryonic fibroblasts followed by PCR amplification of the target sequence and analysis for indel formation by T7 endonuclease 1 digestion as described [31]. The circular pX330 plasmid containing the active *AS160* exon 1 target (5 ng/μl final concentration) [32] was used for pronuclear microinjection of rat zygotes. Fertilized eggs for microinjection were produced by mating superovulated Wistar female rats (Envigo Hsd:WI, Strain Code 001) with Wistar males. Pronuclear microinjection was carried out as described [33]. A total of 297 zygotes were microinjected, 266 surviving zygotes were transferred to pseudopregnant SD female rats (Charles River Laboratory, SAS SD, Strain Code 400), and 58 pups were born. Genotyping of genomic DNA extracted from tail tip biopsies by PCR identified 24 founder animals with indels in *AS160* exon 1.

### Rat genotyping and breeding

Animals were genotyped by PCR with DNA isolated from tail samples using primers flanking the sgRNA binding site: 5′- GGCTGGTGGCACCGAGTCAGG-3′ (forward), 5′- CCGACGGATCTCGGCCATGAG-3′ (reverse) followed by sequencing using a nested primer 5′- GCGCGGTGCCCTCGCTAGGC-3′. A founder line carrying a 20-bp substitution deletion (TGGCGACAAGCGTTCCGGC) was selected for colony expansion and backcrossed with wildtype Wistar outbred strain (Charles River Laboratory; Wilmington, VA) to establish an *AS160*^***+/-***^ colony. Genotyping was performed by Transnetyx (Cordova, TN) using a forward primer 5′-CCTAGCGCAGCCAGGTG-3′ and reverse primer 5′-TCCTGCGATCCAAGCAAGAC-3′ together with reporters 5′-CCGGAAGCGCTTGTC-3′ and 5′-CCGGAAGCGCTTGTC-3′ to detect wildtype and mutant, respectively.

Transgenic animals were backcrossed with a WT Wistar [Crl:WI] background (Charles River Laboratories) a total of 4 times. The AS160-KO rat breeding colony was maintained by the Unit for Laboratory Animal Medicine (ULAM) husbandry services at the University of Michigan. Tail biopsies from 2 weeks-old rats were collected and used for genotyping. Upon weaning, homozygous mutant (AS160-KO) and control WT sibling animals were transferred to a ULAM holding facility and housed with a 12 h light/dark cycle with free access of food (5LOD chow) and water.

### Body mass, body composition and tissue masses

Body composition using an NMR analyzer (Minispec LF9011, Bruker Optics) and body mass were measured in rats at 7–8 weeks-old (n = 6 for each genotype). A different cohort of rats was anesthetized with an intraperitoneal injection of ketamine/xylazine cocktail (50 mg/kg ketamine and 5 mg/kg xylazine) at 11 weeks-old, and body mass was also determined in these rats. In addition, the heart and skeletal muscles (extensor digitorum longus, EDL, epitrochlearis, gastrocnemius, and soleus) were isolated and weighed from these rats.

### Indirect calorimetry, physical activity and food intake

Indirect calorimetry and physical activity were determined in rats (n = 6 for each genotype) at 9 weeks of age using the Comprehensive Lab Animal Monitoring System (CLAMS, Columbus Instruments, Columbus, OH). Rats were housed in the CLAMS unit for 72 hours with ad libitum water and food access. Food intake was also determined for these rats at 10–11 weeks-old.

### Oral glucose tolerance test (OGTT)

Following a 15–16 hour overnight fast, 11 weeks-old male WT (n = 6) and AS160-KO (n = 6) rats were given 50% glucose via oral gavage at 2.0g/kg. Blood samples were collected prior to and after the gavage at time 0, 15, 30, 60, and 120 minutes via the tail vein. Blood levels of glucose were measured using a glucometer (Accu-Chek, Roche), and plasma levels of insulin were determined by ELISA (Millipore Sigma, Burlington, MA). Animals were restrained for less than a minute each time while blood samples were collected. The total area under the curve (AUC) for glucose and for insulin was calculated using the trapezoidal rule [34].

### Hyperinsulinemic-euglycemic clamp (HEC)

Male WT (n = 6) and transgenic (AS160-KO; n = 6) rats had catheters surgically placed into the jugular vein and carotid artery one week prior to a HEC experiment performed when rats were 10 weeks-old as previously described [35]. At approximately 1700 h on the previous day of the clamp experiment, food was removed from rat cages (approximately 16 h prior to the start of the clamp procedure). The protocol consisted of a 90 min tracer equilibration period (t = -90 to 0 min) beginning at ~0930 h, followed by a 120 min experimental period (t = 0 to 120 min) beginning at ~1100 h. At t = -90, a bolus infusion of 12 μCi of [3-^3^H]-glucose (HPLC purified; PerkinElmer) was given, followed by a 0.125 μCi/min infusion for 90 min. At t = -10 min, a blood sample (~100 μl) was taken for assessment of basal levels of insulin and glucose and glucose turnover. The insulin infusion was begun at t = 0 with a primed-continuous infusion (200 mU/kg bolus, followed by 20 mU/kg/min or 120 pmol/kg/min) of human insulin (Novo Nordisk). The infusion of [3-^3^H]-glucose was increased to 0.20 μCi/min for the rest of the experiment to minimize changes of specific activity from the equilibration period. Euglycemia (120–130 mg/dL) was maintained during the clamp by measuring blood glucose every 10 min using an Accu-Chek glucometer (Roche, Germany) starting at t = 0 and infusing 50% glucose at variable rates accordingly. Blood samples (100 μl) were collected during a steady-state of glucose infusion at t = 80, 85, 90, 100, 110 and 120 min for determination of glucose specific activity. Blood insulin concentrations were determined from samples taken at t = -10 and 120 min. To estimate glucose uptake in tissues, a bolus intravenous injection of [^14^C]-2-DG was given at t = 78 minutes while continuously maintaining the HEC steady state. Blood samples were taken at 2, 7, 12, 22, 32 and 42 minutes after the injection for determination of plasma [^14^C]-2-DG radioactivity. At the end of the experiment, rats were anesthetized with an intravenous infusion of sodium pentobarbital (SP). Tissues [epitrochlearis, gastrocnemius, soleus, EDL, WAT (epididymal), and heart] were rapidly excised and freeze-clamped with aluminum tongs cooled in liquid nitrogen and stored at −80°C until later processing. A portion of these tissues were used to determine 2-DG uptake as previously described [36] and another portion was used for immunoblotting (please see below).

### Plasma insulin and non-esterified fatty acids (NEFA)

During the HEC, aliquots of plasma taken at -10 and 120 minutes were used to determine plasma insulin concentration by ELISA (Millipore Sigma, Burlington, MA). Aliquots of plasma taken at -10, 80, 90 and 120 minutes were used to determine NEFA concentration by the manufacturer’s instructions of a colorimetric assay (Wako Diagnostics, Mountain View, CA).

### Isolated skeletal muscle experiments

At 1600 to 1700 on the evening prior to the isolated muscle experiments, food was removed from the rats’ (9 weeks-old) cages. Animals used for insulin stimulation experiments were anesthetized with intraperitoneal injections of SP (50 mg/kg) while rats used for the 5-aminoimidazole-4-carboxamide-1-β-D-ribofuranoside (AICAR) experiment were anesthetized with a ketamine (50 mg/kg)/xylazine (5 mg/kg) cocktail (K/X). In preliminary experiments, we found no significant difference for glucose uptake by isolated muscles dissected from rats anesthetized with SP versus K/X (results not shown). For insulin experiments, while rats were deeply anesthetized, both of their epitrochlearis and soleus muscles were dissected out. Soleus muscles were longitudinally split into two similarly sized strips. Epitrochlearis muscles and soleus muscle strips were placed in vials including the appropriate media, shaken at 50 revolutions per minute and continuously gassed (95% O_2_−5% CO_2_) in a heated (35°C) water bath. Muscles were incubated in vials containing 2 ml Krebs Henseleit Buffer (KHB) supplemented with bovine serum albumin (BSA; 0.1%), 2 mM sodium pyruvate, 6 mM mannitol with or without insulin (500 μU/ml) for 30 minutes. After the first incubation step, muscles were then transferred to second vial containing KHB-BSA with the same insulin concentration as the preceding step along with 1 mM 2-DG (with final specific activity of 2.25 mCi/mmol [^3^H]-2-DG), and 9 mM mannitol (with final specific activity of 0.022 mCi/mmol [^14^C]-mannitol) for 20 minutes. After the second incubation step, muscles were rapidly blotted on filter paper moistened with ice-cold KHB, trimmed, freeze-clamped using aluminum tongs cooled in liquid nitrogen, and stored at -80°C until subsequent processing and analysis. The same incubation protocol was for AICAR experiments, but only the epitrochlearis was used.

### Tissue lysate preparation

One portion from the tissues from the rats after the HEC was weighed and processed for determination of [^14^C]-2-DG accumulation as previously described [36]. Another portion from each of these tissues and the muscles from the ex vivo incubation experiments were weighed, transferred to pre-chilled glass tissue grinding tubes (Kontes, Vineland, NJ), then homogenized in ice-cold lysis buffer (1 ml) using a glass pestle attached to motorized homogenizer (Caframo, Georgian Bluffs, ON). The lysis buffer included TPER supplemented with 1 mM EDTA, 1 mM EGTA, 2.5 mM sodium pyrophosphate, 1 mM sodium vanadate, 1 mM ß-glycerophosphate, 1 μg/ml leupeptin, and 1 mM phenylmethylsulfonyl fluoride. Homogenates were rotated for 1 h (4°C) prior to centrifugation (15,000 x g for 15 minutes at 4°C). The supernatants were transferred to microfuge tubes and stored at -80°C for subsequent analyses. Protein concentration was measured using the bicinchoninic acid procedure according to the manufacturer’s instructions [37].

### Determination of glucose uptake by isolated muscles

Aliquots (200 μl) of the supernatants from centrifuged lysates that were prepared from incubated muscles were combined in a vial with 8 ml of scintillation cocktail (Research Products International, Mount Prospect, IL), and a scintillation counter (Perkin Elmer) was used to determine ^3^H and ^14^C disintegrations per minute. These values were used to determine [^3^H]-2-DG uptake as previously described [38, 39].

### Immunoblotting

An equal amount of protein of each sample was mixed with Laemmli buffer, boiled for 5 minutes and separated using SDS-PAGE (9% resolving gel) before being transferred to polyvinylidene fluoride (PVDF) membranes. Membranes were blocked in 5% BSA or non-fat milk in TBST for 1 h at room temperature and transferred to 5% BSA or milk-TBST with the appropriate primary antibody for overnight at 4°C. Membranes underwent 3 x 5 minutes washes in TBST and were then incubated with secondary antibody in 5% BSA or nonfat milk for 1 h at room temperature. Blots were again washed 3 x 5 minutes in TBST and 2 x 5 minutes TBS. Finally, PVDF membranes were subjected to enhanced chemiluminescence (ECL) to visualize protein bands and immunoreactive proteins were quantified by densitometry (Fluorchem E and AlphaView Imaging Software; ProteinSimple, San Jose, CA). Equal loading was adjusted by Memcode staining and values were normalized to the average of all values on each blot.

### Skeletal muscle myosin heavy chain isoform analysis

Soleus, EDL, epitrochlearis and gastrocnemius muscles of rats (10 weeks-old) were isolated, weighed and processed as previously described for MHC isoform abundance [40]. MHC bands were quantified using densitometry.

### Statistical analysis

Data were statistically analyzed using SigmaPlot 13.0 (San Jose, CA). Comparisons between the two genotypes were performed using an unpaired two-tailed *t*-test. Comparisons between contralateral muscles that were incubated ex vivo (either ±insulin or ±AICAR) were performed with a paired two-tailed *t*-test. Two-way ANOVA was used to compare means among more than two groups (ex vivo muscles incubated ±insulin or ±AICAR), and Holm-Sidak post hoc tests were used to identify the source of significant variance. Data were expressed as means ± SEM. P-values ≤0.05 were considered statistically significant.

## Results

### Confirmation of genotype

**Fig 1:**
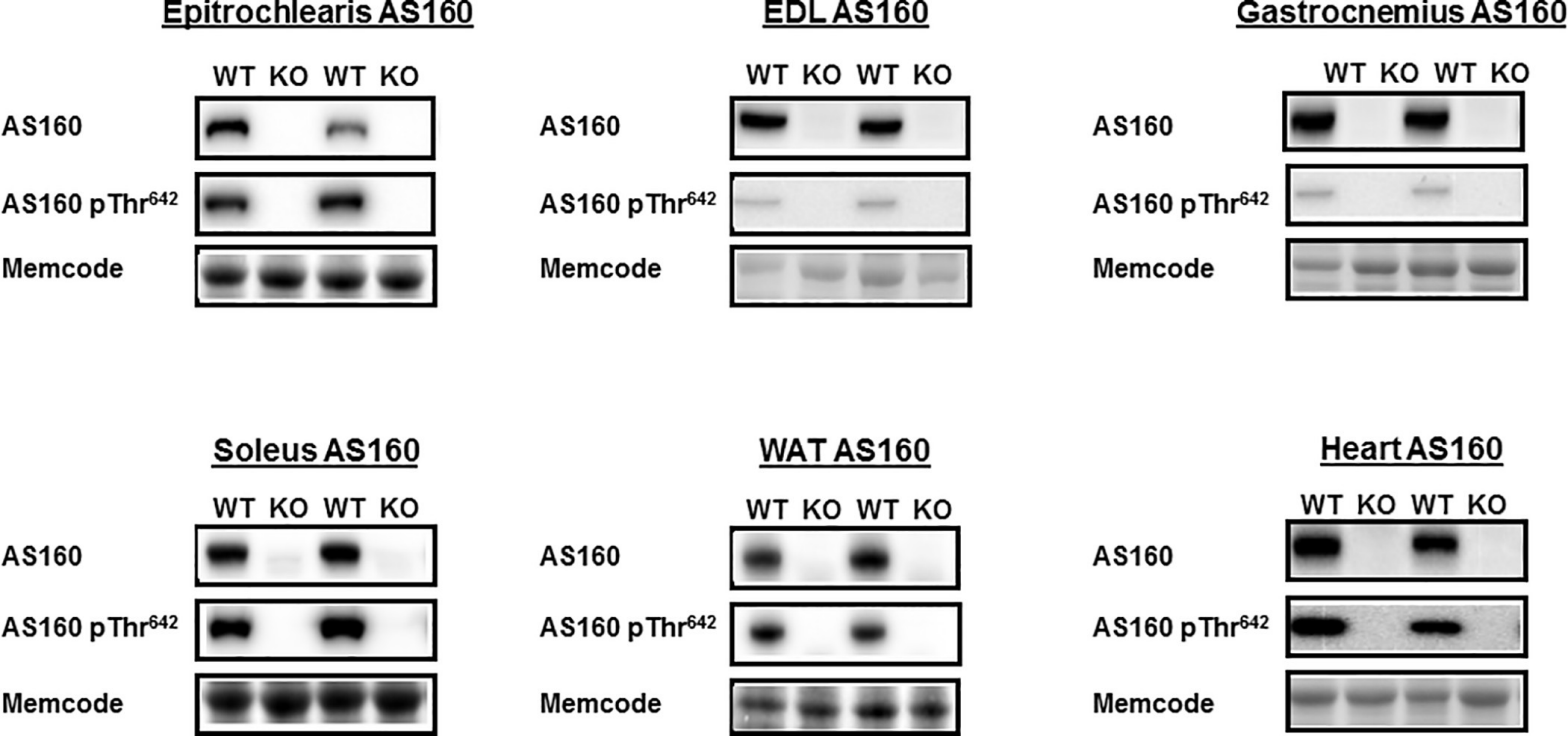
Total AS160 abundance and AS160 phosphorylation (pAS160 Thr^642^) in tissues collected immediately after the hyperinsulinemic-euglycemic clamp performed in WT and KO rats. EDL, extensor digitorum longus; WAT, white adipose tissue. Images of representative immunoblots and loading controls (Memcode protein stain) are provided for each group. Neither total AS160 nor pAS160 Thr^642^ was detectable in any of the tissues from KO rats. N = 5–6 rats per group.

For each rat, genotype was assessed by qPCR analysis using DNA isolated from tail samples. Absence of AS160 in AS160-KO rats was confirmed after the rats were euthanized based on AS160 protein in skeletal muscle assessed by immunoblotting. The same rats were used for both the body composition analysis and HEC, and AS160 protein was determined in these rats in multiple tissues (epitrochlearis, gastrocnemius, EDL, soleus, WAT, and heart, Fig 1).

### Body mass and body composition, and tissue masses

There were no significant genotype-related (WT versus AS160-KO) differences in body mass or body composition (fat, lean and fluid) or masses of skeletal muscles or the heart (Table 1). The skeletal muscle/body mass and heart mass/body mass ratios were also not significantly different between genotypes (data not shown).

**Table 1:** Body mass, body composition and tissue masses.

	WT	KO
**Body Mass, g**	232.57 ±13.37	246.13 ±11.63
**Body Fat Mass, %**	7.30 ±0.67	7.37 ±0.32
**Body Lean Mass, %**	75.49 ±0.50	75.32 ±0.50
**Body Fluid Mass, %**	7.46 ±0.09	7.57 ±0.08
**Extensor digitorum longus, mg**	170.2 ±7.2	170.0 ±7.7
**Epitrochlearis, mg**	85.6 ±7.3	94.8 ±9.5
**Gastrocnemius, mg**	1810.3 ±61.3	1823.4 ±64.5
**Soleus, mg**	192.5 ±7.9	195.5 ±6.5
**Heart, mg**	1059.8 ± 38.0	1141.9 ±33.8

Body composition was determined using an NMR analyzer in rats at 7–8 weeks-old. Skeletal muscles (extensor digitoroum longus, epitrochlearis, gastrocnemius and soleus) and heart from 11-week-old rats were isolated and weighed. Values are means ± SEM (*n* = 6 rats per genotype). WT, wildtype. KO, AS160-KO.

### Skeletal muscle MHC isoform abundance

There were no significant genotype-related differences in muscle fiber type composition based on relative MHC isoform abundance (Table 2) in the EDL, epitrochlearis, gastrocnemius or soleus muscles.

**Table 2:** Relative myosin heavy chain isoform composition of skeletal muscles.

	%MHC-I		%MHC-IIA		%MHC-IIB		%MHC-IIX	
	WT	KO	WT	KO	WT	KO	WT	KO
**EDL**	0	0	8.9 ±1.1	10.1 ±1.0	55.0 ±0.6	52.9 ±4.4	36.1 ±0.5	37.0 ±3.6
**EPI**	5.6 ±1.9	5.9 ±2.1	11.9 ±0.5	9.5 ±1.0	53.1 ±2.0	56.5 ±2.7	29.4 ±2.5	28.1 ±1.0
**GAS**	6.7 ±3.4	7.2 ±2.3	11.1 ±3.6	12.6 ±4.2	43.9 ±3.8	41.2 ±6.0	38.2 ±3.3	39.1 ±7.7
**SOL**	87.9 ±4.4	83.6 ±1.4	12.1 ±4.4	16.4 ±1.4	0	0	0	0

Values are means ±SEM (*n* = 3–6 rats per genotype). WT, wildtype. KO, AS160-KO. Myosin heavy chain (MHC) isoform is expressed as relative values (%) for each of the 4 skeletal muscles: extensor digitorum longus (EDL), epitrochlearis (EPI), gastrocnemius (GAS), and soleus (SOL).

### Indirect calorimetry, physical activity and food intake

AS160-KO and WT rats were not significantly different for oxygen consumption, respiratory exchange ratio (RER), energy expenditure, physical activity (total X-activity, ambulatory X-activity or vertical Z-physical activity) (Table 3) or food intake (WT = 27.7 ±0.8 g/day and AS160-KO = 28.3 ±1.6 g/day).

**Table 3:** Indirect calorimetry and physical activity.

	Dark		Light	
	WT	AS160-KO	WT	AS160-KO
**VO**_**2**_ **(ml/kg/h)**	1652.9 ±73.2	1752.0 ±44.9	1294.9 ±40.0	1330.3 ±24.6
**RER (VCO**_**2**_**/VO**_**2**_**)**	0.954 ±0.004	0.956 ±0.005	0.930 ±0.007	0.945 ±0.011
**Energy Expenditure (kcal/kg/h)**	8.137 ±0.392	8.687 ±0.227	6.392 ±0.199	6.544 ±0.112
**Activity-X Total (counts/h)**	1316.5 ±117.3	1423.4 ±98.8	341.1 ±5.7	395.3 ±34.5
**Activity-X Ambulatory (counts/h)**	505.5 ±58.4	538.5 ±37.0	123.4 ±4.9	130.7 ±10.8
**Activity-Z (counts/h)**	527.4 ±101.8	737.2 ±119.0	107.5 ±21.7	163.7 ±39.7

Values are means ±SEM (*n* = 4–6 rats per genotype). WT, wildtype. KO, AS160-KO. VO_2_, oxygen consumption rate. RER, respiratory exchange ratio. VCO_2_, carbon dioxide production rate.

### Oral glucose tolerance test (OGTT)

**Fig 2:**
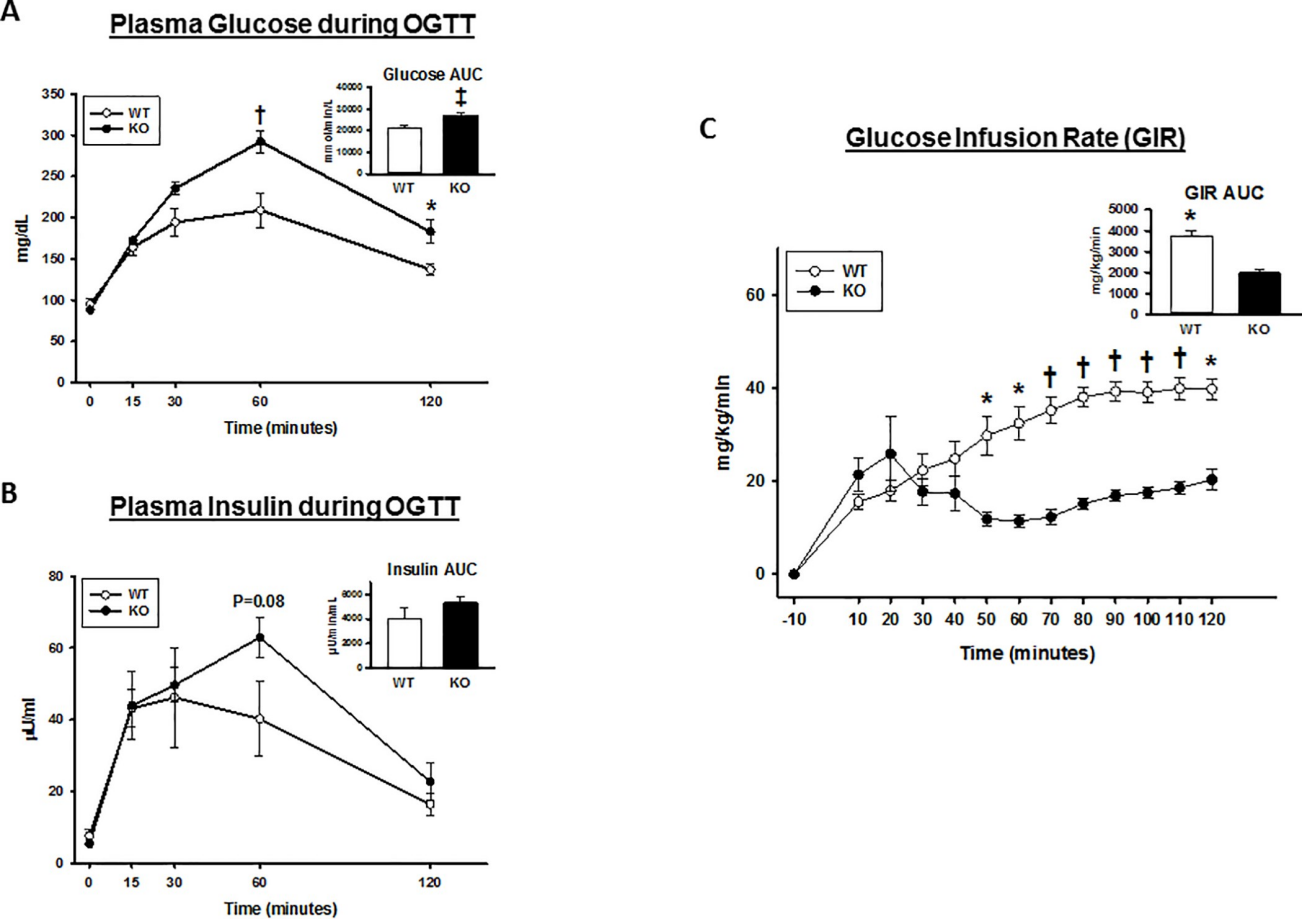
Oral glucose tolerance test (OGTT) and glucose infusion rate (GIR during the hyperinsulinemic-euglycemic clamp; HEC) in wildtype (WT; open bars and open circles) and AS160-KO (KO; filled bars and filled circles) rats. Area under the curve (AUC; inset) was calculated by the trapezoidal method for (A) plasma glucose and (B) plasma insulin. Data were analyzed by Student’s t-test. *P<0.05, ^†^P<0.01 and ^‡^P<0.005 for WT versus KO rats. Values are means ± SEM. N = 6 rats per group. (C) GIR in rats undergoing HEC. AUC for GIR was calculated by the trapezoidal method (inset). Data were analyzed by Student’s t-test. *P<0.05 and ^†^P<0.005 for WT versus KO rats. Values are means ± SEM. N = 5–6 rats per group.

Basal blood glucose concentration was not significantly different for WT versus AS160-KO rats (Fig 2A). AS160-KO rats were glucose intolerant compared to their respective WT controls based on higher glucose values during the OGTT (minutes 60 and 120 minutes; P values ranging from 0.05 to 0.001) and on glucose area under the curve (AUC) for AS160-KO versus WT was significantly greater (p<0.005; Fig 2). Basal plasma insulin concentration did not differ between genotypes, but plasma insulin had a non-significant trend to be greater for AS160-KO versus WT rats at 60 minutes (P = 0.08) (Fig 2B).

### Hyperinsulinemic-euglycemic clamp (HEC)

Prior to the HEC, there were no significant genotype effects on blood glucose concentration, plasma insulin concentration, glucose turnover rate (GTR) or hepatic glucose production (HGP; Table 4). Glycemia during the HEC was modestly, but significantly greater (P<0.05) in WT versus AS160-KO rats. Insulin during the HEC was not significantly different between genotypes (Table 4). Neither HGP nor insulin suppression of HGP were significantly different between genotypes. As expected, the glucose infusion rate (GIR) AUC during the HEC was substantially lower for AS160-KO versus WT rats (45%; P<0.005; Fig 2C). GIR was significantly lower at minutes 50, 60, 70, 80, 90, 100, 110 and 120 (P<0.005 to 0.001). Similarly, GTR during the HEC was markedly lower for AS160-KO versus WT rats (52%; P<0.001). Due to the differential glucose uptake results in the heart, we sought to evaluate non-esterified fatty acids (NEFA) in plasma from the same animals undergoing the HEC. WT rats and their AS160-KO littermates showed no difference in levels of plasma non-esterified fatty acid at the basal period and three time points during the clamp steady state (WT versus AS160-KO: -10 min, 0.612 ±0.043 versus 0.621 ±0.024; 80 min, 0.143 ±0.006 versus 0.148 ±0.004; 90 min, 0.141 ±0.004 versus 0.145 ±0.008; 120 min, 0.141 ±0.005 versus 0.146 ±0.003).

**Table 4:** Hyperinsulinemic euglycemic clamp values.

	WT	KO
**Basal blood glucose, mmol/L**	6.26 ±0.14	5.97 ±0.22
**Clamp blood glucose, mmol/L**	6.75 ±0.21*	5.78 ±0.14
**Basal plasma insulin, μU/mL**	19.59 ±6.47	7.92 ±1.12
**Clamp plasma insulin, μU/mL**	498.01 ±3.58	500.95 ±12.22
**GIR, μmol/kg/min**	219.62 ±10.98^**†**^	101.95 ±7.13
**Clamp GTR, μmol/kg/min**	233.49 ±17.88^**†**^	112.16 ±7.86
**Basal HGP, μmol/kg/min**	44.12 ±3.36	45.67 ±3.81
**Clamp HGP, μmol/kg/min**	13.88 ±9.29	10.20 ±5.30
**Suppression of HGP rate, %**	71.49 ±20.19	80.43 ±11.50

Values means ± SEM (*n* = 5–6 rats per genotype). WT, wildtype. KO, AS160-KO. GIR, glucose infusion rate. GTR, glucose turnover rate. HGP, hepatic glucose production.

*P < 0.05

^**†**^P <0.001, WT versus KO.

### In vivo glucose uptake by tissues during the HEC

**Fig 3:**
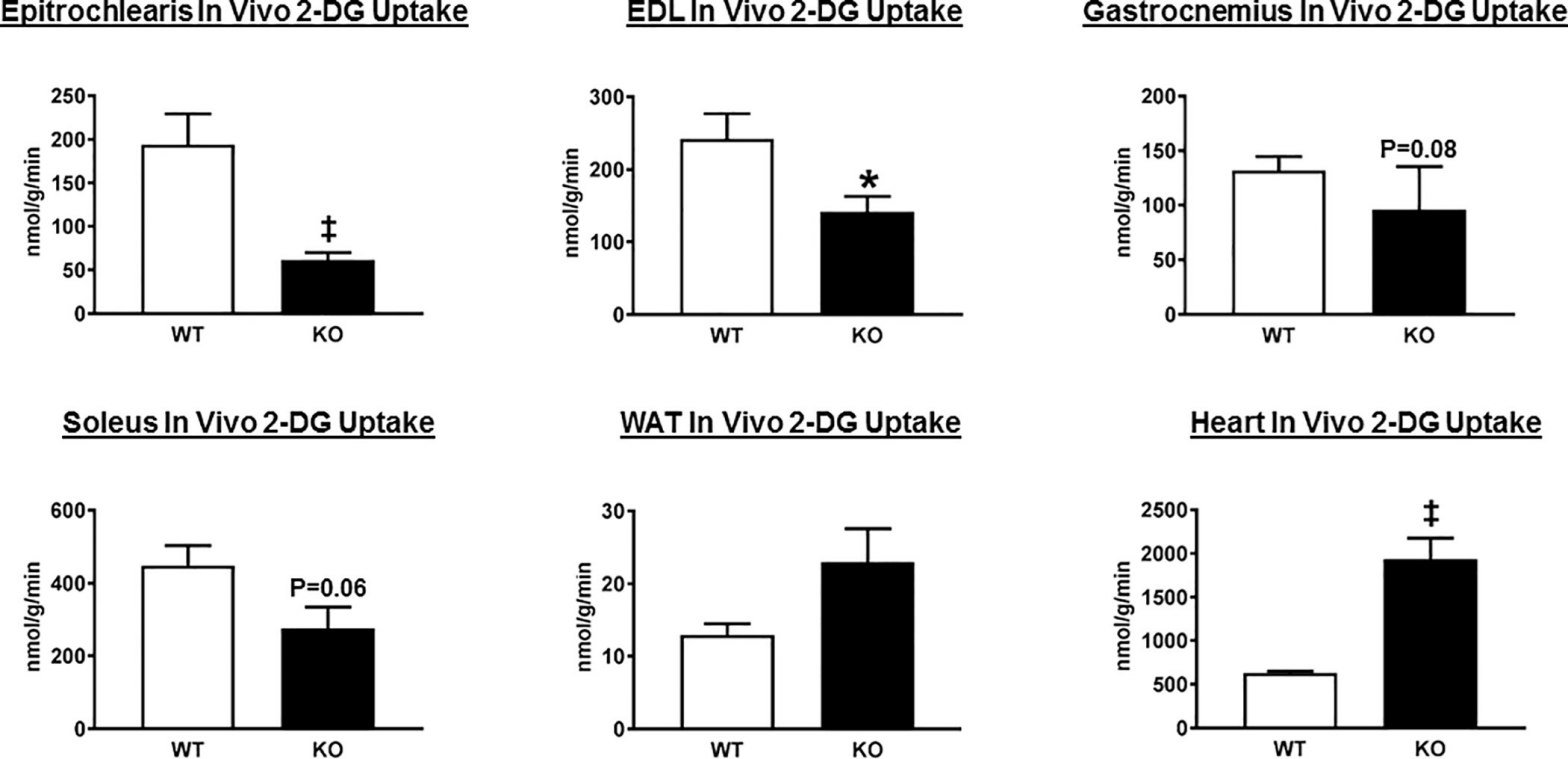
In vivo tissue 2-deoxyglucose (2-DG) uptake in wildtype (WT; open bars) and AS160-KO (KO; filled bars) rats from the hyperinsulinemic-euglycemic clamp experiment. Extensor digitorum longus (EDL); White adipose tissue (WAT). Data were analyzed by Student’s t-test. *P<0.05 and ^‡^P<0.005 for WT versus KO rats. Values are means ± SEM. N = 5–6 rats per group.

AS160-KO compared to WT animals had significantly lower glucose uptake in the epitrochlearis (P<0.01) and in the EDL (P<0.05) (Fig 3). There was a non-significant trend for glucose uptake to be lower for AS160-KO versus WT rats in the gastrocnemius (P = 0.08) and the soleus (P = 0.06). In white adipose tissue (WAT), there was a trend for greater (P = 0.09) glucose uptake in AS160-KO versus WT controls. Myocardial glucose uptake was markedly (approximately 3-fold) greater for AS160-KO compared to WT controls (P<0.005).

### Immunoblotting of tissues from the HEC

**Fig 4:**
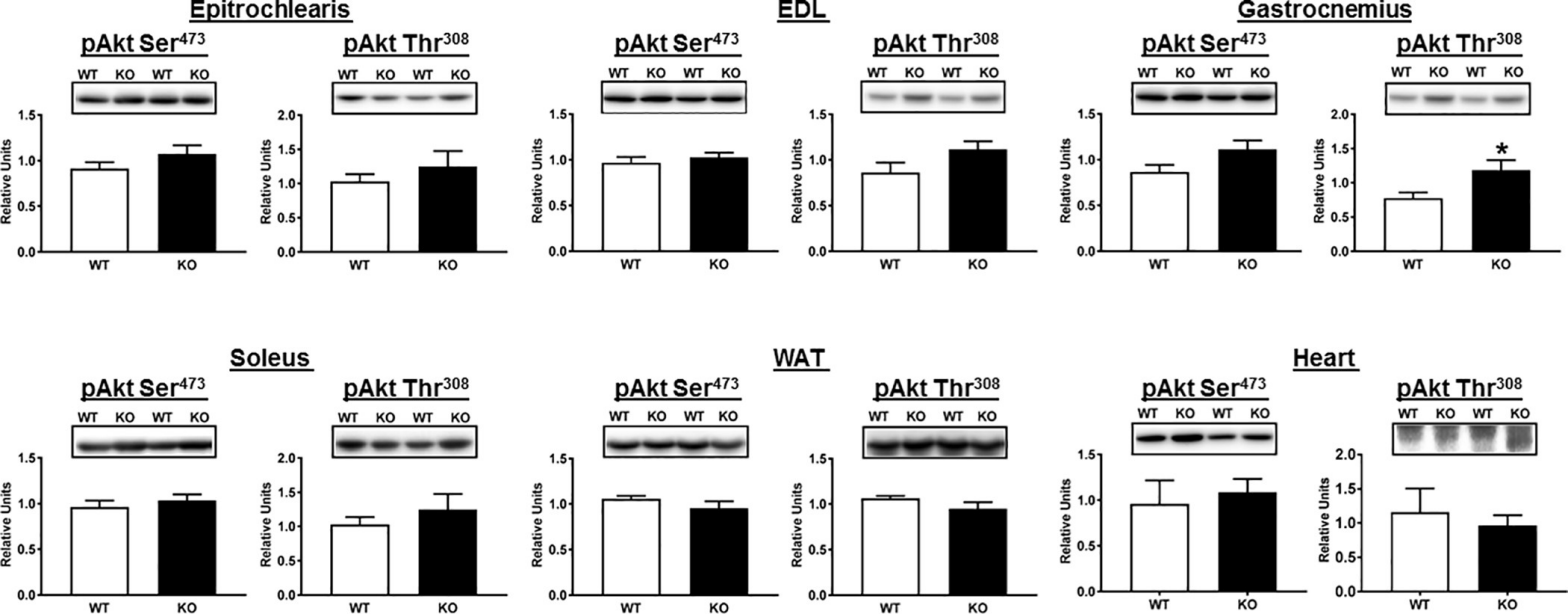
Phosphorylation of Akt on Ser^473^ (pAkt Ser^473^) and Thr^308^ (pAkt Thr^308^) in tissues collected immediately after the hyperinsulinemic-euglycemic clamp performed in WT (open bars) and KO (filled bars) rats. EDL, extensor digitorum longus; WAT, white adipose tissue. Bar graphs represent the ratio of the values for the immunoblots and their respective loading controls (Memcode stain). Because no genotype differences were detected for loading controls, images of the loading controls are not included to improve the clarity of these figures, as well as the other immunoblotting figures that follow. *P<0.05, for WT versus KO rats. Values are means ± SEM. N = 5–6 rats per group.

**Fig 5:**
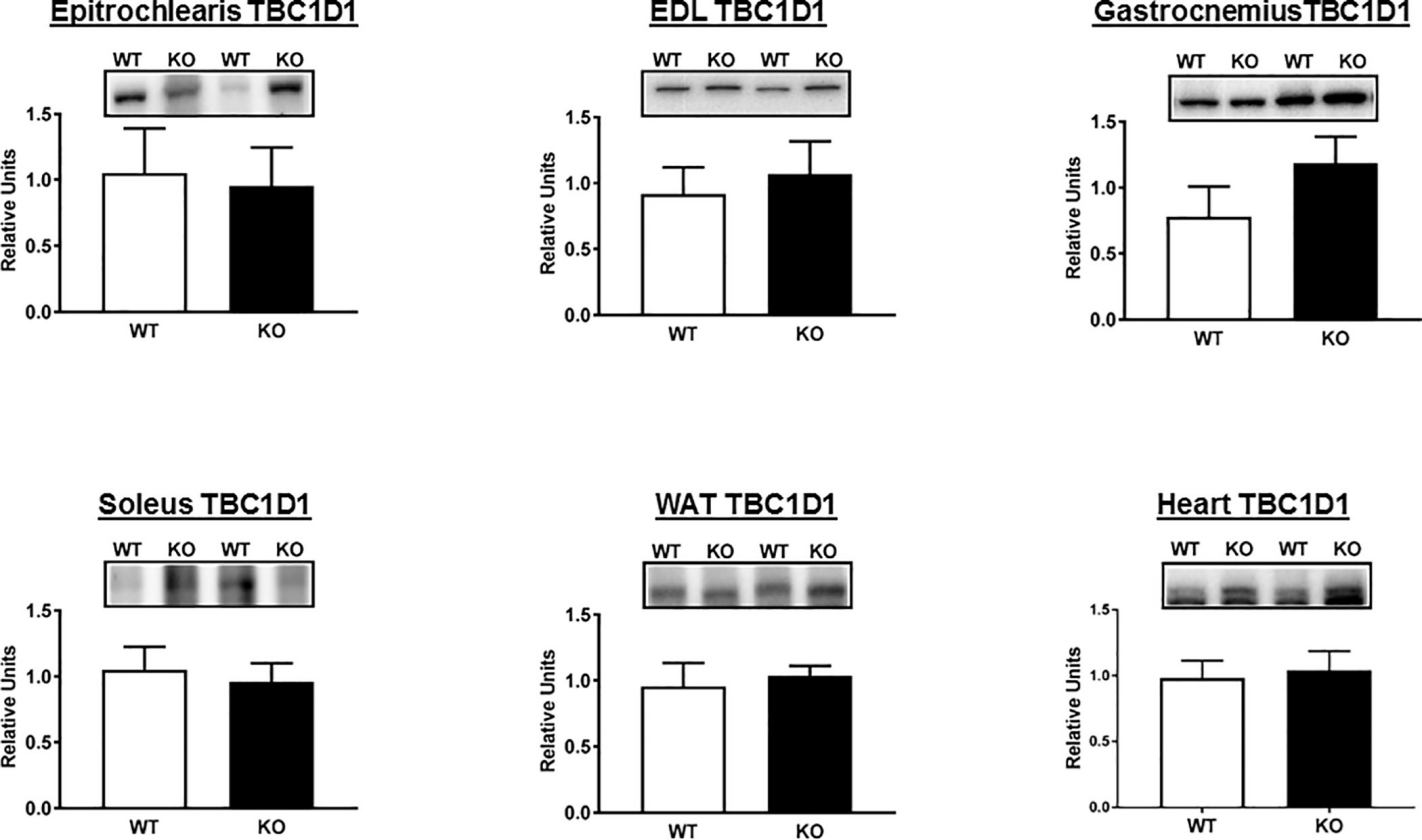
Total abundance TBC1D1 in tissues collected immediately after the hyperinsulinemic-euglycemic clamp performed in WT (open bars) and KO (filled bars) rats. EDL, extensor digitorum longus; WAT, white adipose tissue. Bar graphs represent the ratio of the values for the immunoblots and their respective loading controls (Memcode stain). Values are means ± SEM. N = 5–6 rats per group.

A portion of the tissues sampled for glucose uptake was analyzed by immunoblotting. Thr^642^-AS160 is an Akt-phosphomotif that is essential for the full-effect of insulin on glucose uptake [2, 41]. As expected, both total AS160 protein and pAS160 Thr^642^ were detectable in all of the tissues from WT rats, and they were undetectable in all of the tissues from AS160-KO rats (Fig 1). There were no genotype-related differences for pAkt Ser^473^ in any of the tissues (Fig 4), and the only genotype-related difference for pAkt Thr^308^ was a slightly greater value for AS160-KO versus WT in the gastrocnemius (Fig 4). We conclude that the differences in tissue glucose uptake were not attributable to genotype-related deficits in proximal signaling as evidenced by pAkt. TBC1D1 protein abundance was not significantly different between genotypes for any of the tissues studied (Fig 5).

**Fig 6:**
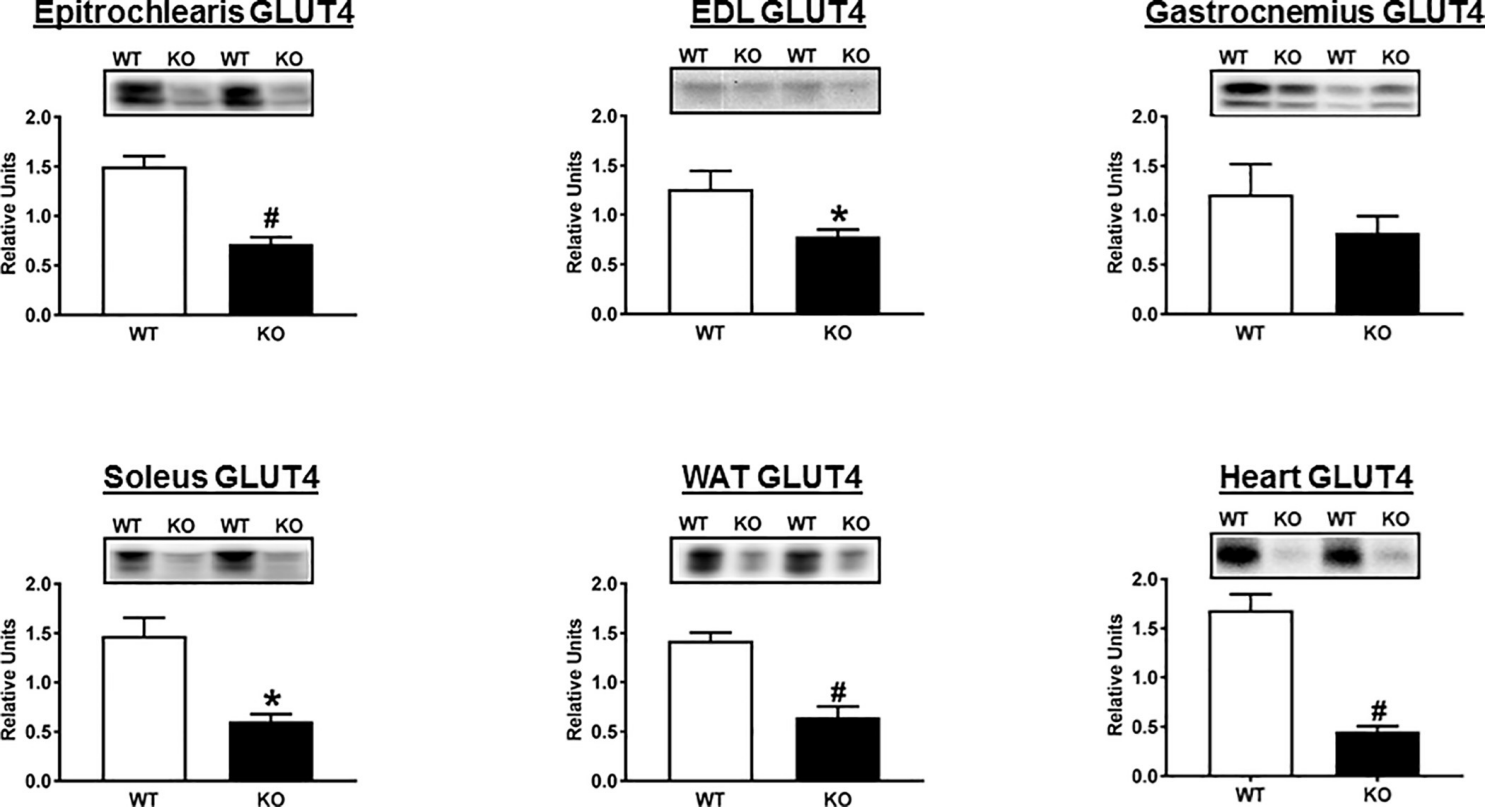
Total abundance of GLUT4 in tissues collected immediately after the hyperinsulinemic-euglycemic clamp performed in WT (open bars) and KO (filled bars) rats. EDL, extensor digitorum longus; WAT, white adipose tissue. Bar graphs represent the ratio of the values for the immunoblots and their respective loading controls (Memcode stain). *P<0.05 and ^#^P<0.001 for WT versus KO rats. Values are means ± SEM. N = 5–6 rats per group.

We also assessed GLUT4, the major glucose transporters expressed by each of the tissues collected after the HEC, and hexokinase II, the predominant enzyme which catalyzes glucose phosphorylation in these tissues. The abundance of GLUT4, the major insulin-regulated glucose transporter, was substantially and significantly lower for the epitrochlearis, EDL, soleus, WAT and heart (Fig 6). GLUT4 values in the gastrocnemius were not significantly different between genotypes. In contrast to GLUT4, there was no evidence for genotype-related differences in either GLUT1 or hexokinase II abundance in any tissue analyzed (data not shown).

**Fig 7:**
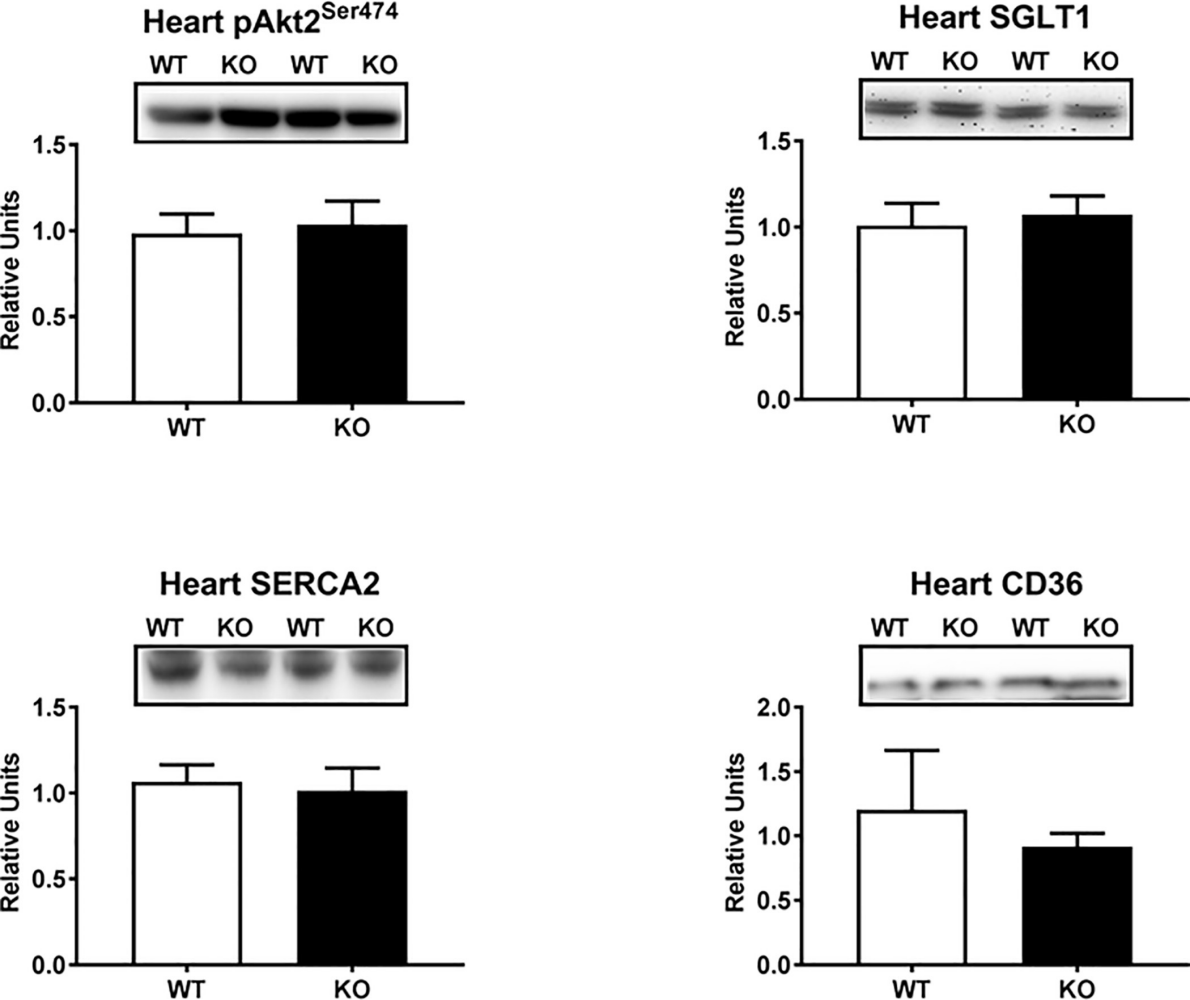
Akt2 phosphorylation on Ser^474^ (pAkt2^Ser474^) and abundance of SGLT1, SERCA2, and CD36 in heart collected immediately after the hyperinsulinemic-euglycemic clamp performed in WT (open bars) and KO (filled bars) rats. Bar graphs represent the ratio of the values for the immunoblots and their respective loading controls (Memcode stain). Values are means ± SEM. N = 5–6 rats per group.

The substantial increase in myocardial glucose uptake in AS160-KO versus WT rats was especially striking given the markedly reduced GLUT4 abundance in the AS160-KO heart. Accordingly, we performed additional analyses to test potential mechanisms that might account for the myocardial glucose uptake results. Both Akt1 and Akt2 are highly expressed by the heart, but only Akt2 is essential for insulin-stimulated myocardial glucose uptake [42]. Therefore, we evaluated both Akt2 abundance and Akt2 phosphorylation on Ser^474^ (pAkt2^Ser474^) in the heart from the rats undergoing the HEC. Neither Akt2 abundance (data not shown) nor pAkt2^Ser474^ (Fig 7) differed between genotypes. Because increased myocardial expression of the sodium-dependent glucose cotransporter 1 (SGLT1) has been reported to be compensatory mechanism in diabetic rodents with reduced GLUT1 and GLUT4 expression in the heart [43], we determined SGLT1 abundance in the hearts collected from WT and AS160-KO rats after the HEC. However, no difference between genotypes was observed for SGLT1 protein abundance (Fig 7). Sarcoplasmic reticulum calcium ATPase (SERCA) has been identified as a positive regulator of glucose transport in the heart [44]. Accordingly, in the hearts sampled from rats after the HEC, we also determined the abundance of SERCA2, which is the SERCA isoform that is highly expressed by the heart. No genotype difference in SERCA2 abundance was observed (Fig 7). The heart has a high capacity for oxidation of both glucose and lipids, and there can be reciprocal relationship between the relative rates of metabolism of these two fuels [45]. Because CD36 is a crucial fatty acid translocase protein that facilitates fatty acid uptake by the heart [46], we also assessed CD36 abundance in the hearts of rats after the HEC. However, no significant difference was found between WT and AS160-KO rats for myocardial CD36 abundance (Fig 7).

### Ex vivo glucose uptake and immunoblotting of insulin-stimulated skeletal muscle

**Fig 8:**
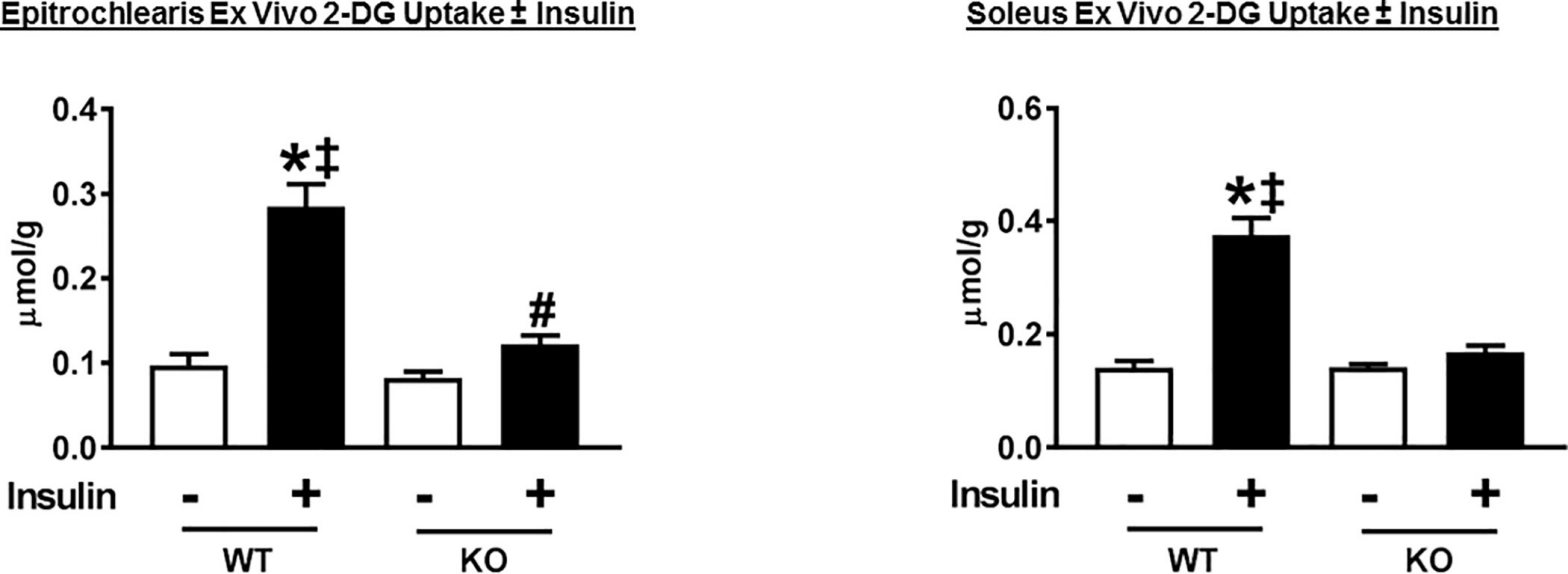
2-Deoxyglucose (2-DG) uptake by ex vivo incubated skeletal muscles (epitrochlearis and soleus) from WT and AS160-KO rats. Paired muscles were incubated without (open bars) or with (filled bars) 500 μU/ml insulin. Data were analyzed by two-way ANOVA, and Holm-Sidak post hoc analysis was used to identify the source of significant variance. *P<0.001 (WT) and ^#^P<0.05 (KO), no insulin versus insulin within the same genotype; ^‡^P<0.001, WT versus KO with the same insulin concentration. A paired *t*-test revealed significantly greater glucose uptake with insulin versus without insulin in the KO soleus (P<0.05). Values are means ± SEM. N = 12–21 rats per group.

Evaluating glucose uptake by isolated muscles provides insights into the tissue’s inherent capacity for glucose uptake, independent of the direct effects of systemic factors. Paired epitrochlearis and soleus muscles were isolated from rats to determine ex vivo glucose with and without insulin using an insulin concentration (500 μU/ml) that was very similar to the plasma insulin concentration for the rats in the HEC. Insulin-stimulated glucose uptake by isolated epitrochlearis (P<0.001) and soleus (P<0.001) from WT rats markedly exceeded values for their AS160-KO controls (Fig 8). In the epitrochlearis of AS160-KO rats, glucose uptake with insulin was greater than glucose uptake values without insulin (P<0.05). A paired *t*-test revealed significantly greater glucose uptake with insulin versus without insulin in the AS160-KO soleus (P<0.05).

**Fig 9:**
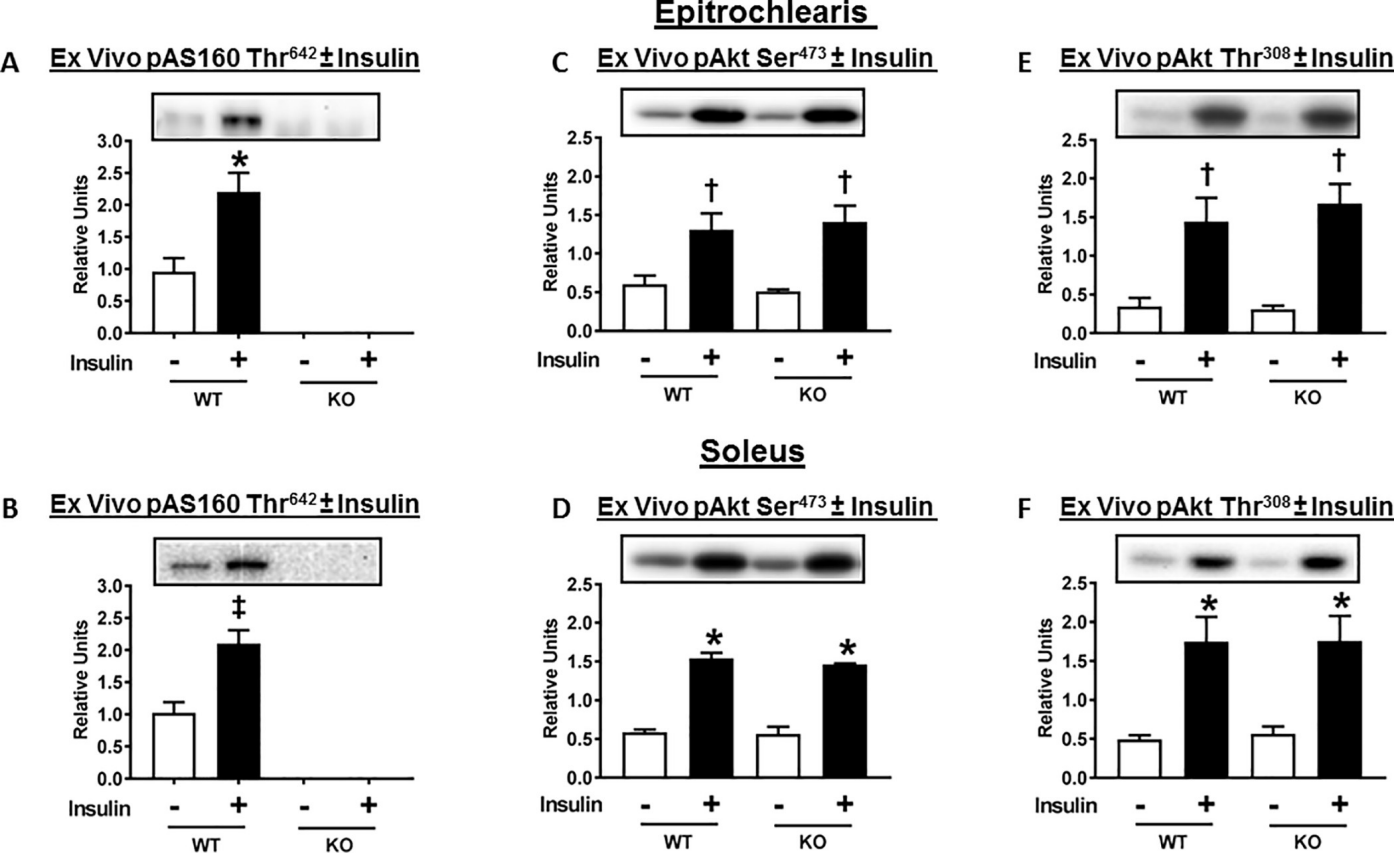
AS160 phosphorylation (pAS160 Thr^642^) and Akt phosphorylation (pAkt Ser^473^ and pAkt Thr^308^) in ex vivo incubated skeletal muscles (epitrochlearis and soleus) from WT and AS160-KO rats. Paired muscles were incubated without (open bars) or with (filled bars) 500 μU/ml insulin. Bar graphs represent the ratio of the values for the immunoblots and their respective loading controls (Memcode stain). Fig 9 (A) (B), AS160 pThr^642^ was undetectable in any of the tissues from KO rats. Data were analyzed by t-test. *P<0.05 and ^‡^P<0.005, no insulin versus insulin in WT rats. Values are means ± SEM. N = 3–7 rats per group. Fig 9 (C) (D) (E) (F), *P<0.001, ^†^P<0.05, no insulin versus insulin in rats with same genotype. Values are means ± SEM. N = 3 rats per group.

As expected, insulin led to marked and significant increase in pAS160 Thr^642^ in both epitrochlearis (P<0.05) and soleus (P<0.005) muscles from WT rats (Fig 9). pThr^642^-AS160 was undetectable in either epitrochlearis or soleus muscles from AS160-KO rats (Fig 9A and 9B). Insulin caused a substantial increase in Akt Ser^473^ and Thr^308^ (Fig 9C–9F) phosphorylation in both epitrochlearis and soleus muscles. No genotype-related differences were detected for insulin-stimulated Akt phosphorylation in either muscle. The genotype-related insulin resistance was not attributable to reduced proximal signaling as indicated by pAkt.

### Ex vivo glucose uptake and immunoblotting of AICAR-stimulated skeletal muscle

**Fig 10:**
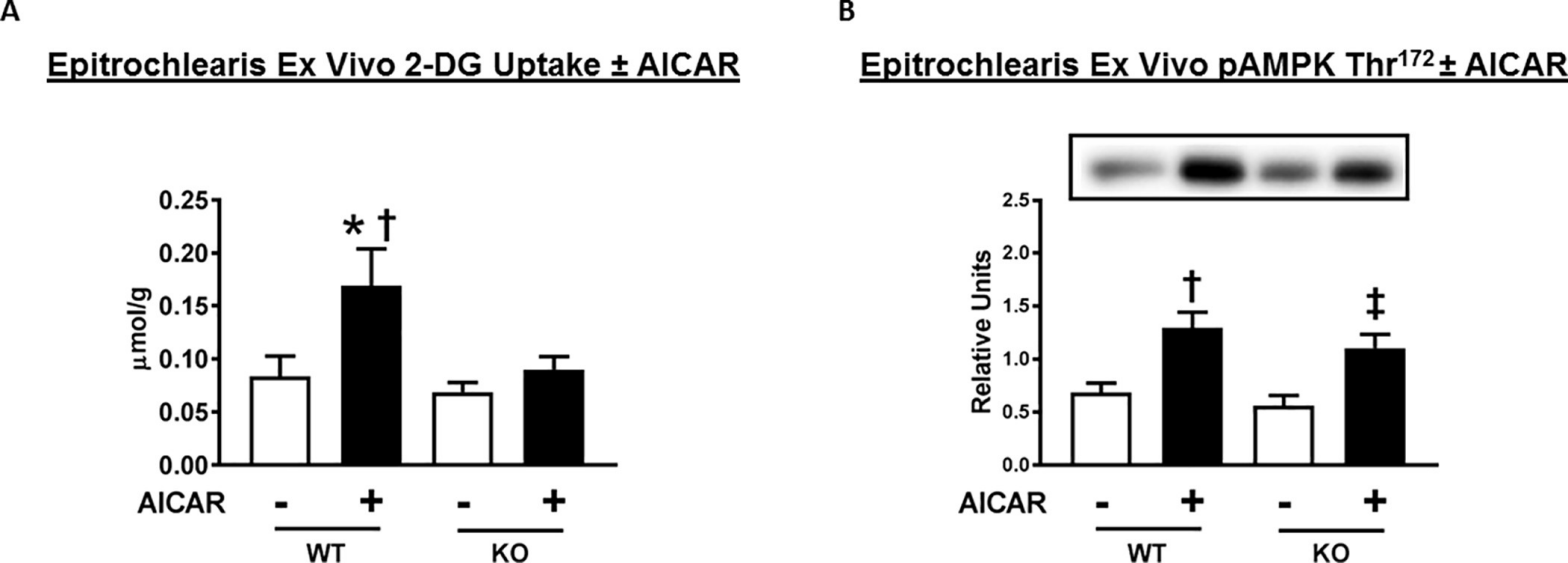
2-Deoxyglucose (2-DG) uptake and phosphorylation of AMP-activated protein kinase (AMPK) on Thr^172^ (pAMPK Thr^172^) in ex vivo incubated epitrochlearis from WT and AS160-KO rats. Paired muscles were incubated without (open bars) or with (filled bars) 2 mM AICAR. Data were analyzed by two-way ANOVA, and Holm-Sidak post hoc analysis was used to identify the source of significant variance. (A) *P<0.01 for no AICAR versus AICAR within the same genotype; ^†^P<0.01 for WT versus KO with the same AICAR concentration. Values are means ± SEM. N = 5–9 rats per group. (B) Bar graphs represent the ratio of the values for the immunoblots and their respective loading controls (Memcode stain). ^†^P<0.01 (in WT) for no AICAR versus AICAR; ^‡^P<0.005 for no AICAR versus AICAR in KO. Values are means ± SEM. N = 5–9 rats per group.

To test if the genotype-related changes in glucose uptake were specific to insulin-dependent mechanisms, we used AICAR, an AMPK activator that can induce greater glucose uptake in the absence of insulin. The soleus was not studied with AICAR because prior research indicated that although AICAR leads to substantial increase in glucose uptake by isolated rat epitrochlearis [47], it does not induce greater glucose uptake by isolated rat soleus [48]. As expected, epitrochlearis muscles incubated in the presence compared to the absence of AICAR had significantly (P<0.01) greater glucose uptake in muscles from WT rats (Fig 10A). Glucose uptake by AICAR-stimulated muscles was substantially greater for WT versus AS160-KO rats (P<0.01). When compared using a paired *t*-test, glucose uptake by muscles from AS160-KO rats incubated with AICAR versus muscles incubated without AICAR were not significantly different (P<0.17).

In WT rats, epitrochlearis muscles incubated with AICAR compared to without AICAR had significantly greater pAMPK Thr^172^ (Fig 10B). There was no significant genotype-related difference in pAMPK Thr^172^. In AS160-KO rats, AICAR-treatment significantly (P<0.05) increased pAMPK Thr^172^.

## Discussion

The physiological characterization of the novel preclinical rat model of AS160 deficiency created in this study provides valuable information about AS160’s role in regulating glucose metabolism. Many aspects of the phenotype (body composition, energy expenditure, whole body insulin sensitivity, and skeletal muscle GLUT4 protein abundance) were quite similar for AS160-KO rats compared to mice and humans with genetic AS160 deficiency [4, 18, 19, 22].

AS160-KO compared to WT rats had greater blood glucose values during an OGTT. AS160-KO mice versus WT controls have been studied with an intraperitoneal GTT (IGTT) rather than an OGTT. The results have indicated either no detectable genotype difference or modest glucose intolerance [18, 19, 22, 23]. Genetic AS160 deficiency in humans is associated with greater 2-h glucose values during an OGTT [4]. Thus, AS160 deficiency can result in modest glucose intolerance in the absence of fasting hyperglycemia in rats and mice, and these results are similar to those in humans with genetic disruption of AS160. AS160 deficiency in humans is linked to substantially increased risk to develop type 2 diabetes (odds-ratio of 10.3) [4]. Diabetes has not been reported in AS160-KO rodents, but these animals have not yet been studied under conditions that might unmask a possible underlying greater risk for developing diabetes, e.g., eating high fat and/or high fructose diet.

AS160-KO versus WT rats were insulin resistant on the basis of lower GIR and GTR values during the HEC. Wang et al. [19] observed a lower GIR in the AS160-KO versus WT mice. Genetic AS160 deficiency in humans is associated with insulin resistance based on glucose and insulin values from an OGTT [4]. Systemic insulin resistance is a hallmark of AS160 deficiency across species.

Lower glucose uptake by skeletal muscle played a major role in their systemic insulin resistance. Glucose uptake during the HEC was substantially lower for AS160-KO compared to WT rats in the epitrochlearis and EDL muscles. The gastrocnemius and soleus also tended to be insulin resistant. In the only study that evaluated skeletal muscle glucose uptake during a HEC in AS160-KO mice, Wang et al. [19] reported insulin resistance for glucose uptake by the vastus lateralis and the soleus of AS160-KO mice. Lansey et al. [18] also found substantially lower in vivo glucose uptake by the gastrocnemius, EDL and tibialis anterior (TA) muscles of AS160-KO mice compared to WT mice injected with radiolabeled 2-DG and glucose to stimulate insulin secretion. Skeletal muscle insulin resistance has not been directly assessed in humans with AS160 deficiency, but lower glucose uptake by most insulin-stimulated skeletal muscles has been consistently found in AS160-KO rodents compared to WT controls.

In vivo assessment of glucose uptake is essential to understand physiological glucose metabolism, but ex vivo muscle preparations provide valuable insights into the inherent capacity for glucose uptake by individual muscles, independent of the immediate effects of systemic factors, including blood flow, circulating factors and muscle recruitment. Isolated epitrochlearis and soleus muscles of AS160-KO rats were profoundly insulin resistant. What accounts for this insulin resistance? It was independent of any genotype-effect on the abundance of hexokinase II protein, a protein that can influence the rate of muscle glucose uptake. The lower glucose uptake was likely secondary, at least in large part, to the markedly lower GLUT4 protein abundance of AS160-KO versus WT rats for both the epitrochlearis and soleus. Lower GLUT4 protein abundance in skeletal muscle of AS160-deficient humans versus their controls [4] would favor attenuated muscle glucose uptake. Lower ex vivo glucose uptake and GLUT4 abundance in the soleus of AS160-KO mice compared to WT controls has also been reported in in mice [18, 19, 22].

AICAR-stimulated glucose uptake was evaluated in the epitrochlearis to determine if lower glucose uptake was specific to insulin-stimulation. The markedly reduced AICAR effect on glucose uptake by the epitrochlearis from AS160-KO rats versus WT controls indicated that the lower insulin-stimulated glucose uptake in AS160-KO rats does not represent an insulin-specific outcome. More likely, the reduced GLUT4 abundance is crucial for lower glucose uptake with either AICAR or insulin in the AS160-KO rats. Chadt et al. [22] reported that both GLUT4 abundance and AICAR-stimulated glucose uptake by isolated soleus muscles were reduced in male AS160-KO versus WT mice. Chadt et al. [22] also reported that neither GLUT4 abundance nor AICAR-stimulated glucose uptake were reduced in the isolated EDL of AS160-KO versus WT mice. Thus, muscles from AS160-KO rodents with markedly reduced GLUT4 abundance (rat epitrochlearis and mouse soleus) also had reduced glucose uptake with either insulin or AICAR, and the muscle from AS160-KO mice that was characterized by no reduction in GLUT4 (EDL) [22], had no reductions in either insulin-stimulated or AICAR-stimulated glucose uptake.

In insulin-stimulated AS160-KO rats compared to WT rats, glucose uptake by WAT during the HEC non-significantly tended to increase even though GLUT4 abundance was substantially lower in the WAT for AS160-KO versus WT rats. Neither Akt phosphorylation nor hexokinase II abundance differed between genotypes in the WAT. Glucose uptake by WAT (epididymal) was not lower in AS160-KO mice versus WT controls after an injection with glucose to stimulate insulin secretion [18]. In contrast to these results, adipose cells isolated from the WAT (epididymal depot) of AS160-KO mice compared to WT controls had lower insulin-stimulated glucose uptake and lower GLUT4 abundance [18, 19, 22]. The reason for no decline in vivo glucose uptake by WAT from AS160-KO versus WT controls remains to be determined, but systemic factors may be responsible.

A striking and unexpected discovery was the ~3-fold greater glucose uptake by the heart of AS160-KO rats versus WT controls. This large increase occurred even though there was a substantial decline in myocardial GLUT4 abundance in AS160-KO versus WT rats. There were no genotype differences in the heart for phosphorylation of Akt, and no genotype-effects on either GLUT1 or hexokinase II abundance. What might account for the marked increase in myocardial glucose uptake? The energetic demands of the heart at rest is normally largely (~50–70%) from fat oxidation with much of the balance of energy derived from carbohydrate [49, 50]. Lansey et al. [18] reported that circulating NEFA concentration of AS160-KO mice exceeded values for WT mice either under basal conditions (fasting for 6 h) or 2 h after a glucose injection that increased insulin. However, NEFA had not been reported for AS160-deficient animals or humans during an HEC. It seemed possible that the greater myocardial glucose uptake might be related to alterations in lipid metabolism. Therefore, we evaluated plasma NEFA values and found no genotype-related differences either before or during the HEC. Although plasma NEFA levels were not different between genotypes, the fatty acid translocase CD36 is a key determinant of myocardial fatty acid uptake, and research on cultured cardiac cells has implicated AS160 as crucial for regulating the subcellular localization of CD36 in response to insulin [51]. However, WT and KO rats did not differ for myocardial CD36 abundance.

Although little has previously been reported about AS160’s functional roles in the heart, there is evidence that AS160 can influence myocardial electrocardiographic properties. Mice with a knockin mutation of AS160 that replaced Thr649 (equivalent to Thr642) with alanine versus WT controls were characterized by moderately increased R-wave amplitude [52]. Greater R-wave amplitude is used to diagnose myocardial hypertrophy, and myocardial hypertrophy and heart failure is associated with greater reliance on glucose for energy [49, 53]. However, there was no evidence for myocardial hypertrophy in KO versus WT rats based on heart mass or heart mass/body mass ratio. We also evaluated several other proteins that have been linked to increased myocardial glucose uptake, but no genotype-related differences were observed in pAkt2^Ser473^, SGLT1 abundance or SERCA2 abundance. Additional research is needed to elucidate the mechanisms for AS160’s effects on glucose uptake in the heart.

In conclusion, the creation of a novel AS160-KO rat model coupled with substantial metabolic phenotyping revealed that for many outcomes previously reported in mice and/or humans, AS160 deficiency had comparable consequences in rats. A notable and unexpected discovery was markedly greater myocardial glucose uptake in AS160-KO compared to WT rats in spite of reduced myocardial GLUT4 protein abundance without alterations in circulating NEFA concentration, myocardial phosphorylation of total Akt or Akt2, or the abundance of several proteins that can influence glucose uptake by the heart, including GLUT1, hexokinase II, SGLT1 and SERCA2. In addition to enabling these unexpected results for myocardial glucose uptake, the AS160-KO rat model has the potential to be useful for other avenues of physiological research in which AS160 is implicated as a key regulatory protein, including altered skeletal muscle insulin-stimulated glucose uptake in response to high fat diets, calorie restriction or acute and chronic exercise.

## References

[1] Cartee GD. Roles of TBC1D1 and TBC1D4 in insulin- and exercise-stimulated glucose transport of skeletal muscle. Diabetologia. 2015;58(1):19–30. 10.1007/s00125-014-3395-5 25280670 PMC4258142

[2] Sano H, Kane S, Sano E, Miinea CP, Asara JM, Lane WS, et al Insulin-stimulated phosphorylation of a Rab GTPase-activating protein regulates GLUT4 translocation. J Biol Chem. 2003;278(17):14599–602. 10.1074/jbc.C300063200 . 12637568

[3] Leto D, Saltiel AR. Regulation of glucose transport by insulin: traffic control of GLUT4. Nat Rev Mol Cell Biol. 2012;13(6):383–96. 10.1038/nrm3351 . 22617471

[4] Moltke I, Grarup N, Jorgensen ME, Bjerregaard P, Treebak JT, Fumagalli M, et al A common Greenlandic TBC1D4 variant confers muscle insulin resistance and type 2 diabetes. Nature. 2014;512(7513):190–3. 10.1038/nature13425 . 25043022

[5] Arias EB, Kim J, Funai K, Cartee GD. Prior exercise increases phosphorylation of Akt substrate of 160 kDa (AS160) in rat skeletal muscle. Am J Physiol Endocrinol Metab. 2007;292(4):E1191–200. 10.1152/ajpendo.00602.2006 . 17179389

[6] Bruss MD, Arias EB, Lienhard GE, Cartee GD. Increased phosphorylation of Akt substrate of 160 kDa (AS160) in rat skeletal muscle in response to insulin or contractile activity. Diabetes. 2005;54(1):41–50. . 15616009 10.2337/diabetes.54.1.41

[7] Castorena CM, Arias EB, Sharma N, Cartee GD. Postexercise improvement in insulin-stimulated glucose uptake occurs concomitant with greater AS160 phosphorylation in muscle from normal and insulin-resistant rats. Diabetes. 2014;63(7):2297–308. 10.2337/db13-1686 24608437 PMC4066340

[8] Funai K, Schweitzer GG, Sharma N, Kanzaki M, Cartee GD. Increased AS160 phosphorylation, but not TBC1D1 phosphorylation, with increased postexercise insulin sensitivity in rat skeletal muscle. Am J Physiol Endocrinol Metab. 2009;297(1):E242–51. Epub 2009/05/14. 10.1152/ajpendo.00194.2009 19435856 PMC2711658

[9] Zheng X, Cartee GD. Insulin-induced Effects on the Subcellular Localization of AKT1, AKT2 and AS160 in Rat Skeletal Muscle. Scientific reports. 2016;6:39230 10.1038/srep39230 27966646 PMC5155274

[10] Kramer HF, Witczak CA, Fujii N, Jessen N, Taylor EB, Arnolds DE, et al Distinct Signals Regulate AS160 Phosphorylation in Response to Insulin, AICAR, and Contraction in Mouse Skeletal Muscle. Diabetes. 2006;55(7):2067–76. . 16804077 10.2337/db06-0150

[11] Kramer HF, Witczak CA, Taylor EB, Fujii N, Hirshman MF, Goodyear LJ. AS160 regulates insulin- and contraction-stimulated glucose uptake in mouse skeletal muscle. J Biol Chem. 2006;281(42):31478–85. 10.1074/jbc.M605461200 . 16935857

[12] Kjobsted R, Munk-Hansen N, Birk JB, Foretz M, Viollet B, Bjornholm M, et al Enhanced Muscle Insulin Sensitivity After Contraction/Exercise Is Mediated by AMPK. Diabetes. 2017;66(3):598–612. 10.2337/db16-0530 . 27797909

[13] Pehmoller C, Brandt N, Birk JB, Hoeg LD, Sjoberg KA, Goodyear LJ, et al Exercise alleviates lipid-induced insulin resistance in human skeletal muscle-signaling interaction at the level of TBC1 domain family member 4. Diabetes. 2012;61(11):2743–52. 10.2337/db11-1572 22851577 PMC3478539

[14] Treebak JT, Frosig C, Pehmoller C, Chen S, Maarbjerg SJ, Brandt N, et al Potential role of TBC1D4 in enhanced post-exercise insulin action in human skeletal muscle. Diabetologia. 2009;52(5):891–900. Epub 2009/03/03. 10.1007/s00125-009-1294-y . 19252894 PMC3627047

[15] Karlsson HK, Zierath JR, Kane S, Krook A, Lienhard GE, Wallberg-Henriksson H. Insulin-Stimulated Phosphorylation of the Akt Substrate AS160 Is Impaired in Skeletal Muscle of Type 2 Diabetic Subjects. Diabetes. 2005;54(6):1692–7. . 15919790 10.2337/diabetes.54.6.1692

[16] Consitt LA, Van Meter J, Newton CA, Collier DN, Dar MS, Wojtaszewski JF, et al Impairments in site-specific AS160 phosphorylation and effects of exercise training. Diabetes. 2013;62(10):3437–47. 10.2337/db13-0229 23801578 PMC3781473

[17] DeFronzo RA, Jacot E, Jequier E, Maeder E, Wahren J, Felber JP. The effect of insulin on the disposal of intravenous glucose. Results from indirect calorimetry and hepatic and femoral venous catheterization. Diabetes. 1981;30(12):1000–7. . 7030826 10.2337/diab.30.12.1000

[18] Lansey MN, Walker NN, Hargett SR, Stevens JR, Keller SR. Deletion of Rab GAP AS160 modifies glucose uptake and GLUT4 translocation in primary skeletal muscles and adipocytes and impairs glucose homeostasis. Am J Physiol Endocrinol Metab. 2012;303(10):E1273–86. 10.1152/ajpendo.00316.2012 23011063 PMC3517634

[19] Wang HY, Ducommun S, Quan C, Xie B, Li M, Wasserman DH, et al AS160 deficiency causes whole-body insulin resistance via composite effects in multiple tissues. Biochem J. 2013;449(2):479–89. 10.1042/BJ20120702 23078342 PMC3685216

[20] Lamont BJ, Andrikopoulos S, Funkat A, Favaloro J, Ye JM, Kraegen EW, et al Peripheral insulin resistance develops in transgenic rats overexpressing phosphoenolpyruvate carboxykinase in the kidney. Diabetologia. 2003;46(10):1338–47. 10.1007/s00125-003-1180-y . 12898008

[21] Kraegen EW, James DE, Storlien LH, Burleigh KM, Chisholm DJ. In vivo insulin resistance in individual peripheral tissues of the high fat fed rat: assessment by euglycaemic clamp plus deoxyglucose administration. Diabetologia. 1986;29(3):192–8. . 3516775 10.1007/BF02427092

[22] Chadt A, Immisch A, de Wendt C, Springer C, Zhou Z, Stermann T, et al "Deletion of both Rab-GTPase-activating proteins TBC1D1 and TBC1D4 in mice eliminates insulin- and AICAR-stimulated glucose transport [corrected]. Diabetes. 2015;64(3):746–59. 10.2337/db14-0368 . 25249576

[23] Hargett SR, Walker NN, Keller SR. Rab GAPs AS160 and Tbc1d1 play nonredundant roles in the regulation of glucose and energy homeostasis in mice. Am J Physiol Endocrinol Metab. 2016;310(4):E276–88. 10.1152/ajpendo.00342.2015 26625902 PMC4888528

[24] Xie B, Chen Q, Chen L, Sheng Y, Wang HY, Chen S. The Inactivation of RabGAP Function of AS160 Promotes Lysosomal Degradation of GLUT4 and Causes Postprandial Hyperglycemia and Hyperinsulinemia. Diabetes. 2016;65(11):3327–40. 10.2337/db16-0416 . 27554475

[25] Mackrell JG, Cartee GD. A novel method to measure glucose uptake and myosin heavy chain isoform expression of single fibers from rat skeletal muscle. Diabetes. 2012;61(5):995–1003. 10.2337/db11-1299 22396201 PMC3331778

[26] Pataky MW, Arias EB, Cartee GD. Measuring Both Glucose Uptake and Myosin Heavy Chain Isoform Expression in Single Rat Skeletal Muscle Fibers. Methods Mol Biol. 2019;1889:283–300. 10.1007/978-1-4939-8897-6_17 . 30367421

[27] Cong L, Ran FA, Cox D, Lin S, Barretto R, Habib N, et al Multiplex genome engineering using CRISPR/Cas systems. Science. 2013;339(6121):819–23. 10.1126/science.1231143 23287718 PMC3795411

[28] Mali P, Yang L, Esvelt KM, Aach J, Guell M, DiCarlo JE, et al RNA-guided human genome engineering via Cas9. Science. 2013;339(6121):823–6. 10.1126/science.1232033 23287722 PMC3712628

[29] Popp MW, Maquat LE. Leveraging Rules of Nonsense-Mediated mRNA Decay for Genome Engineering and Personalized Medicine. Cell. 2016;165(6):1319–22. 10.1016/j.cell.2016.05.053 27259145 PMC4924582

[30] Ran FA, Hsu PD, Wright J, Agarwala V, Scott DA, Zhang F. Genome engineering using the CRISPR-Cas9 system. Nat Protoc. 2013;8(11):2281–308. 10.1038/nprot.2013.143 24157548 PMC3969860

[31] Sakurai T, Watanabe S, Kamiyoshi A, Sato M, Shindo T. A single blastocyst assay optimized for detecting CRISPR/Cas9 system-induced indel mutations in mice. BMC Biotechnol. 2014;14:69 10.1186/1472-6750-14-69 25042988 PMC4118159

[32] Mashiko D, Fujihara Y, Satouh Y, Miyata H, Isotani A, Ikawa M. Generation of mutant mice by pronuclear injection of circular plasmid expressing Cas9 and single guided RNA. Scientific reports. 2013;3:3355 10.1038/srep03355 24284873 PMC3842082

[33] Filipiak WE, Saunders TL. Advances in transgenic rat production. Transgenic Res. 2006;15(6):673–86. 10.1007/s11248-006-9002-x . 17009096

[34] Allison DB, Paultre F, Maggio C, Mezzitis N, Pi-Sunyer FX. The use of areas under curves in diabetes research. Diabetes Care. 1995;18(2):245–50. . 7729306 10.2337/diacare.18.2.245

[35] Sharma N, Castorena CM, Cartee GD. Tissue-specific responses of IGF-1/insulin and mTOR signaling in calorie restricted rats. PLoS One. 2012;7(6):e38835 10.1371/journal.pone.0038835 22701721 PMC3368930

[36] Ayala JE, Bracy DP, McGuinness OP, Wasserman DH. Considerations in the design of hyperinsulinemic-euglycemic clamps in the conscious mouse. Diabetes. 2006;55(2):390–7. . 16443772 10.2337/diabetes.55.02.06.db05-0686

[37] Smith PK, Krohn RI, Hermanson GT, Mallia AK, Gartner FH, Provenzano MD, et al Measurement of protein using bicinchoninic acid. Anal Biochem. 1985;150(1):76–85. . 3843705 10.1016/0003-2697(85)90442-7

[38] Cartee GD, Bohn EE. Growth hormone reduces glucose transport but not GLUT-1 or GLUT-4 in adult and old rats. Am J Physiol. 1995;268(5 Pt 1):E902–9. 10.1152/ajpendo.1995.268.5.E902 . 7762644

[39] Hansen PA, Gulve EA, Holloszy JO. Suitability of 2-deoxyglucose for in vitro measurement of glucose transport activity in skeletal muscle. J Appl Physiol. 1994;76(2):979–85. 10.1152/jappl.1994.76.2.979 . 8175614

[40] Castorena CM, Mackrell JG, Bogan JS, Kanzaki M, Cartee GD. Clustering of GLUT4, TUG, and RUVBL2 protein levels correlate with myosin heavy chain isoform pattern in skeletal muscles, but AS160 and TBC1D1 levels do not. Journal of applied physiology. 2011;111(4):1106–17. Epub 2011/07/30. 10.1152/japplphysiol.00631.2011 21799128 PMC3191788

[41] Chen S, Wasserman DH, MacKintosh C, Sakamoto K. Mice with AS160/TBC1D4-Thr649Ala knockin mutation are glucose intolerant with reduced insulin sensitivity and altered GLUT4 trafficking. Cell Metab. 2011;13(1):68–79. Epub 2011/01/05. 10.1016/j.cmet.2010.12.005 . 21195350 PMC3081066

[42] DeBosch B, Sambandam N, Weinheimer C, Courtois M, Muslin AJ. Akt2 regulates cardiac metabolism and cardiomyocyte survival. J Biol Chem. 2006;281(43):32841–51. 10.1074/jbc.M513087200 16950770 PMC2724003

[43] Szablewski L. Glucose transporters in healthy heart and in cardiac disease. Int J Cardiol. 2017;230:70–5. 10.1016/j.ijcard.2016.12.083 . 28034463

[44] Waller AP, Kalyanasundaram A, Hayes S, Periasamy M, Lacombe VA. Sarcoplasmic reticulum Ca2+ ATPase pump is a major regulator of glucose transport in the healthy and diabetic heart. Biochim Biophys Acta. 2015;1852(5):873–81. 10.1016/j.bbadis.2015.01.009 . 25615793

[45] Stanley WC, Recchia FA, Lopaschuk GD. Myocardial substrate metabolism in the normal and failing heart. Physiol Rev. 2005;85(3):1093–129. 10.1152/physrev.00006.2004 . 15987803

[46] Glatz JFC, Luiken J. Dynamic role of the transmembrane glycoprotein CD36 (SR-B2) in cellular fatty acid uptake and utilization. J Lipid Res. 2018;59(7):1084–93. 10.1194/jlr.R082933 29627764 PMC6027920

[47] Wright DC, Hucker KA, Holloszy JO, Han DH. Ca(2+) and AMPK Both Mediate Stimulation of Glucose Transport by Muscle Contractions. Diabetes. 2004;53(2):330–5. . 14747282 10.2337/diabetes.53.2.330

[48] Wright DC, Geiger PC, Holloszy JO, Han DH. Contraction- and hypoxia-stimulated glucose transport is mediated by a Ca2+-dependent mechanism in slow-twitch rat soleus muscle. Am J Physiol Endocrinol Metab. 2005;288(6):E1062–6. 10.1152/ajpendo.00561.2004 . 15657088

[49] Lopaschuk GD, Ussher JR, Folmes CD, Jaswal JS, Stanley WC. Myocardial fatty acid metabolism in health and disease. Physiol Rev. 2010;90(1):207–58. 10.1152/physrev.00015.2009 . 20086077

[50] Abel ED. Glucose transport in the heart. Front Biosci. 2004;9:201–15. . 14766360 10.2741/1216

[51] Samovski D, Su X, Xu Y, Abumrad NA, Stahl PD. Insulin and AMPK regulate FA translocase/CD36 plasma membrane recruitment in cardiomyocytes via Rab GAP AS160 and Rab8a Rab GTPase. J Lipid Res. 2012;53(4):709–17. 10.1194/jlr.M023424 22315395 PMC3307647

[52] Quan C, Xie B, Wang HY, Chen S. PKB-Mediated Thr649 Phosphorylation of AS160/TBC1D4 Regulates the R-Wave Amplitude in the Heart. PLoS One. 2015;10(4):e0124491 10.1371/journal.pone.0124491 25923736 PMC4414484

[53] Abel ED, Kaulbach HC, Tian R, Hopkins JC, Duffy J, Doetschman T, et al Cardiac hypertrophy with preserved contractile function after selective deletion of GLUT4 from the heart. J Clin Invest. 1999;104(12):1703–14. 10.1172/JCI7605 10606624 PMC409881

